# Identification of Potential Sepsis Therapeutic Drugs Using a Zebrafish Rapid Screening Approach

**DOI:** 10.3390/life14121689

**Published:** 2024-12-20

**Authors:** Mark Widder, Chance Carbaugh, William van der Schalie, Ronald Miller, Linda Brennan, Ashley Moore, Robert Campbell, Kevin Akers, Roseanne Ressner, Monica Martin, Michael Madejczyk, Blair Dancy, Patricia Lee, Charlotte Lanteri

**Affiliations:** 1 Walter Reed Army Institute of Research, Silver Spring, MD 20910, USA; 2 General Dynamics Information Technology, Falls Church, VA 22042, USA; 3 Culmen International, LLC, Alexandria, VA 22314, USA; 4 Audie L. Murphy VA Medical Center, San Antonio, TX 78229, USA

**Keywords:** sepsis, endotoxicity, drug discovery, zebrafish, lipopolysaccharide

## Abstract

In the military, combat wound infections can progress rapidly to life-threatening sepsis. The discovery of effective small-molecule drugs to prevent and/or treat sepsis is a priority. To identify potential sepsis drug candidates, we used an optimized larval zebrafish model of endotoxicity/sepsis to screen commercial libraries of drugs approved by the U.S. Food and Drug Administration (FDA) and other active pharmaceutical ingredients (APIs) known to affect pathways implicated in the initiation and progression of sepsis in humans (i.e., inflammation, mitochondrial dysfunction, coagulation, and apoptosis). We induced endotoxicity in 3- and 5-day post fertilization larval zebrafish (characterized by mortality and tail fin edema (vascular leakage)) by immersion exposure to 60 µg/mL *Pseudomonas aeruginosa* lipopolysaccharide (LPS) for 24 h, then screened for the rescue potential of 644 selected drugs at 10 µM through simultaneous exposure to LPS. After LPS exposure, we used a neurobehavioral assay (light-dark test) to further evaluate rescue from endotoxicity and to determine possible off-target drug side effects. We identified 29 drugs with > 60% rescue of tail edema and mortality. Three drugs (Ketanserin, Tegaserod, and Brexpiprazole) produced 100% rescue and did not differ from the controls in the light-dark test, suggesting a lack of off-target neurobehavioral effects. Further testing of these three drugs at a nearly 100% lethal concentration of *Klebsiella pneumoniae* LPS (45 µg/mL) showed 100% rescue from mortality and 88–100% mitigation against tail edema. The success of the three identified drugs in a zebrafish endotoxicity/sepsis model warrants further evaluation in mammalian sepsis models.

## 1. Introduction

The emergence of multidrug-resistant (MDR) bacteria is a global problem for current antibiotic therapies, a difficult challenge for hospitals worldwide, and a threat to effective treatment of infections among combat-wounded service members. Combat wound infections can progress rapidly to life-threatening sepsis, with a median time of three days from injury to the onset of sepsis [1]. A total of 5278 sepsis hospitalizations were recorded in the active component of the U.S. military from 2011 to 2020 [2]. A recent review of the Department of Defense Trauma Registry from 2007 to 2020 found that patients with combat injury-related wound infections who were diagnosed with sepsis had significantly higher mortality rates than non-septic patients (21.9% vs. 4.3%, *p* < 0.001) [3].

The discovery and development of therapeutics that mitigate against host-directed mechanisms responsible for triggering sepsis, to be administered in combination with antibiotics, is a viable research and development path for improving options available for preventing morbidity and mortality associated with sepsis. Sepsis is caused by a dysregulated host response to infection, involving a complex (and not entirely understood) pathophysiology that leads to life-threatening organ dysfunction [4]. Because the mortality rate of sepsis patients continues to be comparatively high relative to non-septic patients, there is an urgent need for improved sepsis therapeutics that specifically address systemic infection and sepsis remediation, which can include modulation of the host response. Small molecule drugs that mitigate against dysregulated host responses to bacterial infection may provide the required systemic approach needed to prevent or treat sepsis.

One problem with identifying new drugs or drug combinations for wound infection and sepsis is the lack of a preclinical model that has better predictive capability than cell-based systems but with higher throughput and lower cost than mammalian models [5]. A whole animal model such as the zebrafish allows for the determination of drug bioactivity, toxicity, and off-target side effects early in the drug discovery process [6], thus filling a gap between in vitro or in silico testing and pre-clinical rodent testing. Zebrafish models of sepsis offer an opportunity for establishing a low-cost and rapid screen to accelerate discovery of potential new small molecule therapies to prevent or treat sepsis. Pathophysiological symptoms observed in septic humans, such as dysregulated inflammatory responses (cytokine storms), tachycardia, endothelial leakage, and progressive edema have been observed in zebrafish infected with bacteria [7]. Zebrafish have a high degree of genetic homology with humans [8] and have been used as a model for a range of human diseases [9]. Zebrafish have demonstrated success in rapid drug screening studies [10]; Ref. [11] note that “Numerous drug treatments that have recently entered the clinic or clinical trials have their genesis in zebrafish”.

Zebrafish are well suited for studies of bacterial infection and sepsis. Zebrafish have been widely used for studying host-pathogen interactions and have an innate and adaptive immune system that become active at about two days and three weeks post-fertilization, respectively [5]. The zebrafish immune system has many similarities to mammals [12,13,14,15,16,17,18], and zebrafish have been used in infection models for bacterial species of particular concern to the military, e.g., *Klebsiella pneumoniae* [19,20] and *Pseudomonas aeruginosa* [21,22,23].

In both mammals and zebrafish, the lipopolysaccharide (LPS) component of the cell wall in gram-negative bacteria can trigger inflammatory responses [24].

To identify potential sepsis therapeutic drugs, a zebrafish endotoxicity/sepsis model [5] was first optimized by evaluating the best combination of endpoints (mortality, tail edema, and reactive oxygen species (ROS) production), LPS sources and concentrations, and exposure times to evaluate the potential of the test drugs to provide rescue from sepsis symptoms. After testing with model compounds previously shown to have efficacy in the zebrafish endotoxicity model, the optimized larval zebrafish model was used to screen the rescue potential of 644 compounds known to affect pathways implicated in the initiation and progression of sepsis in humans. These compounds were derived from commercially available libraries identified to have already been approved by the U.S. Food and Drug Administration (FDA) or other international regulatory agencies for other purposes. Following LPS exposure, a neurobehavioral assay (light-dark test) was used to determine possible off-target drug side effects. Top rescue drugs were tested at a nearly 100% lethal concentration of *K. pneumoniae* LPS (45 µg/mL) to further evaluate rescue potential. The drug candidates with the best ability to rescue zebrafish larvae from LPS-induced endotoxicity without neurobehavioral effects are candidates for further evaluation in mammalian sepsis models.

## 2. Materials and Methods

### 2.1. Zebrafish Husbandry

Adult *Danio rerio* (zebrafish AB strain) from in-house breeding stocks were used to produce high quality embryos used for testing. The zebrafish stocks are outcrossed periodically with new stocks of AB zebrafish obtained from the Zebrafish International Resource Center (Eugene, OR, USA). Zebrafish were maintained at an Association for Assessment and Accreditation of Laboratory Animal Care International (AAALAC) approved facility at the Walter Reed Army Institute for Research (WRAIR). All experiments were performed in compliance with AAALAC guidelines. The zebrafish colony was maintained on a 14-h light and 10-h dark photoperiod under full spectrum LED lighting. The adult zebrafish colony was housed in either flow-through aquaculture racks or in tanks with the following water quality conditions: water temperature 27.5 ± 1.5 °C; dissolved oxygen 7.52 ± 0.3 mg/L; pH 7.56 ± 0.3; alkalinity 110 to 180 mg/L as CaCO_3_; hardness 150 to 210 mg/L as CaCO_3_; conductivity 651 ± 75 µS/cm; and total ammonia less than 0.1 mg/L as NH_3_. Adult zebrafish were fed three times daily on weekdays: two feedings of Gemma Micro 300 (Skretting Zebrafish, Westbrook, ME, USA) and one feeding of live brine shrimp nauplii (Brine Shrimp Direct, Ogden, UT, USA). On weekends, adult zebrafish received two feedings: one Gemma Micro 300 and one live brine shrimp nauplii. Zebrafish aged 6–18 months were bred in I-SPWAN-S breeding chambers (Techniplast, West Chester, PA, USA) to supply embryos for drug screening. Zebrafish embryos from the spawning events were collected in glass petri dishes containing freshly prepared embryo media (EM, see Supplemental Data for formulation). At the conclusion of the study, all surviving larvae were euthanized with sodium hypochlorite according to the AVMA Guidelines for Euthanasia of Animals [25].

### 2.2. Zebrafish Model Optimization: LPS and Endpoint Screening

The endotoxicity/sepsis zebrafish model used was based upon previously published methods [5]. In this model, larval zebrafish (3 days post fertilization (dpf)) were exposed to *Escherichia coli* 0111:B4 LPS by immersion in microplate wells for 24 h, and three endpoints were measured: mortality, tail edema, and ROS production. To facilitate rapid and repeatable drug screening for rescue from LPS-induced endotoxicity, the responsiveness of these three endpoints to alternative LPS sources was determined.

**Figure 1:**
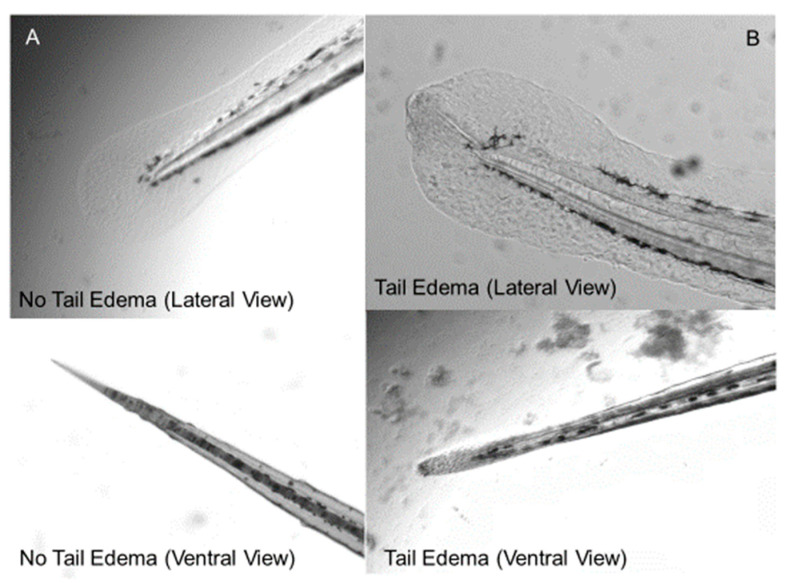
Caudal fin morphology of 3 dpf zebrafish. (**A**) Normal of caudal fin of control fish with no LPS exposure. (**B**) Caudal fin after 24 h of 60 µg/mL of *P. aeruginosa* LPS resulting in a tail edema and vascular leakage.

Three sources of LPS were evaluated: *E. coli* 0111:B4, *P. aeruginosa* Sigma L9143, and *K. pneumoniae*. *E. coli* 0111:B4 LPS was selected as the reference LPS used previously by others [5]. *P. aeruginosa* LPS is commonly associated with infections in hospitals, accounting for an estimated 32,600 cases and resulting in an estimated 2700 deaths in 2017 [26]. *P. aeruginosa* infections are also a common cause of sepsis in both patients with severe burns and immunocompromised patients. *K. pneumoniae* LPS has military relevance related to battlefield injury infections [27]. Initial range finding LC50 tests with larval zebrafish were conducted for each LPS strain at both 3 dpf and 5 dpf. For the 5 dpf LC50 range find test, 15 zebrafish embryos were exposed in 6 well microplates containing 3.5 mL of 0, 25, 50, 100, 150, and 200 µg/mL of LPS in EM. (*E. coli* 0111:B4 was also tested at higher concentrations (400, 800, and 1000 µg/mL) due a lack of toxicity within the initial concentration range). Well plates were then covered with parafilm and placed into an incubator at 28.5 °C for 24 h. After 6 and 24 h, the plates were removed and scored for mortality, tail edema, and ROS production. The tail edema endpoint evaluates vascular leakage from the caudal fin in response to LPS exposure (Figure 1). Tail edema identification (presence/absence) was performed manually by visual inspection using a Nikon SMZ 1270 stereomicroscope with oblique coherent contrast illumination. Initial LPS dose studies were scored by multiple investigators to confirm the accuracy of the method with 100% agreement of scoring. With proper training and familiarization, a 96-well plate was read in approximately 3 to 5 min for both tail edema and mortality. All results reported were scored by the same individual. ROS production was determined as described by Philip et al. (2017) [5] by adding 2,7-dichloroflurescein diacetate (100 µM) to the wells containing the zebrafish larvae and incubating them in a 100% dark photoperiod incubator at 28.5 °C for one hour. Fluorescent images of the incubated zebrafish were then captured with a Keyence BZ-X710 fluorescent microscope using a 2X objective, a BZ-X Filter GFP and a fixed 1/15 s exposure for all imaging. The 3 dpf LC50 range find was the same as the 5 dpf range find with the following changes: 96 well plates were used in place of the 6 well plates, the number of zebrafish was increased from 15 to 32, and the concentration ranges tested for *P. aeruginosa* and *K. pneumoniae* were changed to 10, 20, 30, 40, 50, 60, 70, 80, 90, and 100 µg/mL due to the steep mortality curve in the 5 dpf range find. Results from the LPS toxicity testing and endpoint screening were used to develop the optimized testing procedure through evaluation in selected model compounds, as described in the next section and in the results.

### 2.3. Model Compound Testing

The optimized zebrafish sepsis model was evaluated against four compounds shown to have efficacy in previous zebrafish sepsis models: fasudil, hydrocortisone, dexamethasone, and fisetin. Fasudil, an anti-vascular leakage compound (inhibitor of the RhoA/Rho-kinase pathway) was shown to rescue zebrafish from LPS-induced mortality, ROS production, and tail fin edema [5]. Fasudil also rescued LPS-induced vascular leakage in murine models of experimental sepsis [28] and reduced acute lung injury in septic rats through inhibition of the Rho/ROCK signaling pathway [29]. Hsu et al. (2018) found that the corticosteroids hydrocortisone and dexamethasone produced responses similar to a protein tyrosine phosphatase inhibitor (Shp2 (Ptpn11a) inhibitor 11a-1), leading to decreased inflammatory cytokines, increased *il-10* anti-inflammatory activity, reduced tissue damage and preservation of vascular junction proteins in their zebrafish model [30]. Fisetin is a dietary flavonoid that showed inhibition of LPS-induced inflammation and reduced mortality from endotoxic shock in zebrafish larvae; possible mechanisms include crosstalk between GSK-3β/β-catenin and the NF-κB signaling pathways [31]. Endpoints evaluated with model compounds included mortality, tail edema, and ROS production at 6- and 24-h post exposure to varying concentration ranges of LPS.

### 2.4. Custom Drug Library Selection

Four commercially available drug discovery libraries including nearly 2500 compounds were selected to evaluate drug targets known to affect pathways implicated in the initiation and progression of sepsis in humans [32,33,34,35] including inflammation, mitochondrial dysfunction, coagulation, and apoptosis. Libraries selected included the Selleckchem Cytokine Inhibitor Library (L9500), the Selleckchem Highly Selective Inhibitor Library (L3500), the MedChemExpress Mitochondria-Targeted Compound Library (HY-L089), and the MedChemExpress Coagulation and Anti-coagulation Compound Library (HY-L136). To reduce the high costs associated with developing new or novel drugs, this initial set of candidate compounds was cross-referenced with Selleckchem FDA-Approved Drug Library (L1300) and MedChemExpress FDA-Approved Drug Library (HY-L022). This evaluation identified 644 drugs for sepsis efficacy screening in the optimized zebrafish endotoxicity/sepsis model.

### 2.5. Initial Drug Screening

Healthy, normally developing embryos collected from the adult AB zebrafish spawning events were treated with 75 µM of 1-phenyl 2-thiourea (PTU) in EM prior to 24 h post fertilization (hpf) to block pigmentation formation [36]. These embryos were then placed into an incubator at 28.5 °C until they reached 3 dpf. Embryos were then removed from the incubator and plated individually into the wells of 96 well plates containing 50 µL of EM. A super stock of 120 µg/mL of *P. aeruginosa* LPS was made up in EM in a glass container. This stock was then used to make sub stocks of 120 µg/mL *P. aeruginosa* LPS (positive control) and 120 µg/mL *P. aeruginosa* LPS containing 20 µM of test drug in glass scintillation vials. Immediately after the sub stocks were made, the wells were dosed with either 50 µL of 120 µg/mL of *P. aeruginosa,* 120 µg/mL of *P. aeruginosa* with 20 µM of test drug, or EM (1:1 Dilution). Each 96 well plate had one LPS positive control (*n* = 16), four different test drugs with *P. aeruginosa* LPS (n = 16 per drug), and one negative control (n = 16). Once the plates were dosed, they were covered with parafilm and placed into an incubator at 28.5 °C. The plates were removed at 6- and 24-h post exposure to be scored for tail edema and mortality. All 644 drugs were evaluated for rescue from mortality and tail edema induced by *P. aeruginosa* LPS exposure as compared to LPS-treated controls after 24 h of exposure.

### 2.6. Top Rescue Drug Confirmatory Testing and Down Selection

To further down select the top rescue drugs, only drugs that had >60% rescue of mortality and tail edema induced by *P. aeruginosa* LPS exposure from the initial screen were further evaluated in confirmatory testing. Confirmatory testing followed the same methods as the initial testing but used 5 dpf larvae instead of the 3 dpf larvae that were used for the initial screening because they were easier to handle and replicated the methods of Philip et al. (2017) [5]. The 5 dpf larvae have more fully developed organ functionality than 3 dpf larvae, and in a meta-analysis of acute toxicity data for 600 chemicals, Ducharme et al. (2015) found that larval zebrafish acute toxicity data were predictive of rodent and rabbit acute toxicity for 4 and 5 dpf larvae, but not for 3 dpf larvae [37].

In a second stage of confirmatory testing, drugs that had >80% rescue of mortality and tail edema with the 5 dpf larvae were evaluated further. First, a concentration range find test was used to determine the potential therapeutic window for the drugs. Briefly, 5 dpf zebrafish embryos were placed into the wells of the 96 well plate (one embryo per well), with each well containing 50 µL of EM. Then, an additional 50 µL of either 120 µg/mL of *P. aeruginosa* or 120 µg/mL of *P. aeruginosa* with the test drug in EM (1:1 dilution) was added to the 96 well plate. Each 96 well plate had one positive control (n = 16) and four different concentrations of the test drug with *P. aeruginosa* LPS (*n* = 16 per concentration). Once the plates were dosed, they were covered with parafilm and placed into an incubator at 28.5 °C. The plates were removed at 6- and 24-h post exposure to be scored for tail edema and mortality. The drug concentrations tested were 10, 1, 0.1, 0.01 and 0.001 µM.

Drugs identified with >80% rescue were subjected to additional testing. Drug pretreatment for 24 h prior to LPS challenge was evaluated to determine if any prophylactic benefit could be realized with any of the top rescue drugs. The intent was to determine if drug pre-treatment might be used at the point of injury as a preventative therapeutic before the onset of infection and development of sepsis. Parallel groups of fish received a 24-h drug pre-treatment or no drug pre-treatment prior to LPS challenge. For this test, 4 dpf PTU-treated zebrafish embryos were placed into 96 well plates containing 100 µL of EM. The 24-h drug pre-treatment group had a 10 µM concentration of test drug added when the zebrafish embryos were 4 dpf to allow for a 24-h pre-treatment prior to LPS exposure at 5 dpf. A parallel group that did not receive 24-h drug pre-treatment was evaluated to provide comparative results due to the slight change in LPS challenge/toxicity required to complete the studies. All plates were covered with parafilm and placed into an incubator at 28.5 °C for 24 h. After 24 h, all treatments had an additional 100 µL of either 120 µg/mL of *P. aeruginosa*, 120 µg/mL of *P. aeruginosa* with test drug (to maintain a 10 µM final in well drug concentration and 60 µg/mL of *P. aeruginosa* LPS), or EM alone (control group). Once the plates were dosed, they were covered with parafilm and placed into an incubator at 28.5 °C. The plates were removed at 6- and 24-h post exposure and scored for tail edema and mortality. Once the 24-h tail edema and mortality endpoints were collected, a neurobehavioral test was conducted as described in Section 2.8.

The top 3 drugs (Ketanserin, Tegaserod, and Brexpiprazole) identified from the confirmatory testing were also tested against another strain of LPS to determine if the candidate drug showed efficacy in more than one LPS strain. These tests followed the same methods described in the 5 dpf zebrafish confirmatory testing, but *K. pneumoniae* LPS (45 µg/mL final in well concentration) was used instead of *P. aeruginosa* LPS.

### 2.7. Neurobehavioral Testing

All drugs that were identified with >80% rescue were evaluated at the end of the study using a neurobehavioral assay to evaluate deviations from normal fish behavior and to further evaluate rescue from sepsis, as well as to determine if any of the drugs might be exhibiting off-target side effects. This assay is a modified version of the larval zebrafish light dark transition assay with an added UV light phase (wavelength = 295 nm) to capture any compound-specific neurological effects to UV light. [38,39,40]. The neurobehavioral assay consisted of three phases: a 3-min light phase (white light), a 3-min dark phase (no light) and a 5 s UV phase (UV light only). The light period assesses deviations from normal locomotor behavior, while the rapid transition to dark identifies deviations from normal heightened locomotor activity in this phase. The neurobehavioral assay used a custom trial control script created by Noldus Ethovision XT 15, and it was executed in a Noldus DanioVision system. Zebrafish larvae in microplates were allowed to habituate in the DanioVision system for 5 min under white light conditions prior to the start of the test. The DanioVision system captured larval zebrafish movement throughout this assay at 30 frames per second.

### 2.8. Statistical Analysis

LC50 point estimates and associated 95% confidence intervals for the LPS tests were determined using the Trimmed Spearmen-Karber method [41]. The tail edema and mortality data from the initial and confirmatory drug testing were analyzed using GraphPad Prism 9 using two-sided Fisher’s Exact Test. The two-sided Fisher’s Exact test was chosen as the appropriate statical analysis method over the chi-square test due to the smaller sample sizes for higher throughput initial drug screening (n = 16 fish per treatment). Each drug treatment group was compared pairwise with the on-board positive control group on each test plate. Statistical significance was determined with values of *p* ≤ 0.05. Neurological behavioral testing data was analyzed by Ethovision 15 software that produced an activity analysis profile per frame for each well of the 96 well plates. Any dead embryos were removed from the data set, and the remaining data set was imported into Excel. The offset function in Excel was used to average the percentage of pixel change per 0.1 s for each plate. These averages were then used to calculate the area under the curve (AUC) for each concentration and each phase of the neurological behavioral tests. The behavioral data was then statistically analyzed by drug per phase using a two tailed t-test to determine statistical significance *p* ≤ 0.01. Statistical significance for behavioral endpoints was determined at *p* ≤ 0.01 instead of *p* ≤ 0.05 due to the increased interindividual differences in locomotion activity recorded in the behavioral testing.

## 3. Results

### 3.1. Model Optimization

**Figure 2:**
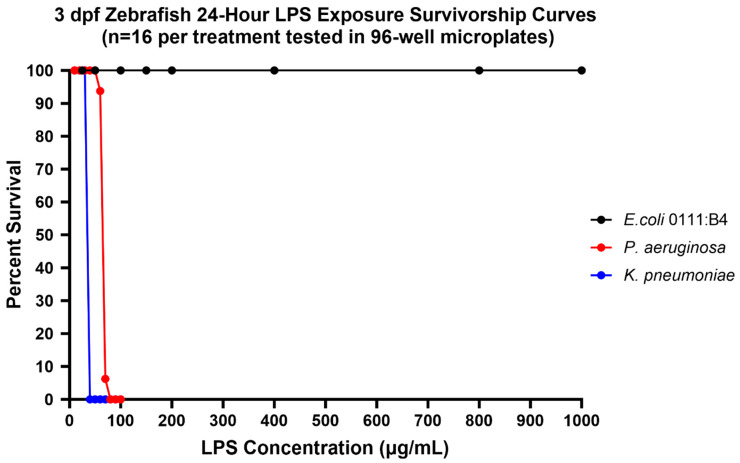

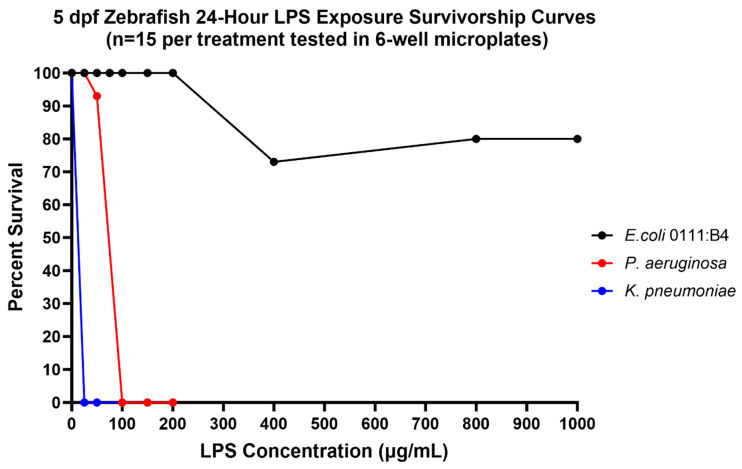
Dose response curves for 3 dpf zebrafish (**top graph**) and 5 dpf zebrafish (**bottom graph**) for 24-h exposures to LPS derived from *E. coli* 0111:B4, *P. aeruginosa*, and *K. pneumoniae*.

Of the three bacterial strains of LPS tested, *K. pneumoniae* LPS was the most toxic, with a 24-h exposure 5 dpf zebrafish LC50 of <25 µg/mL and a 24-h exposure 3 dpf zebrafish LC50 of 34.6 µg/mL (95% confidence limits could not be calculated due to the steepness of the toxicity curve). The second most toxic LPS tested was *P. aeruginosa* with a 24-h exposure to 5 dpf zebrafish LC50 of 67.5 µg/mL (95% confidence limits 61.8–73.8 µg/mL) and a 24-h exposure to 3 dpf zebrafish LC50 of 64.71 µg/mL (95% confidence limits of 63.0–66.5 µg/mL). *E. coli* LPS was over an order of magnitude less toxic to zebrafish with an LC50 of >1000 ug/mL for both 24-h exposures to the 5 dpf and the 3 dpf zebrafish embryos (Figure 2).

**Figure 3:**
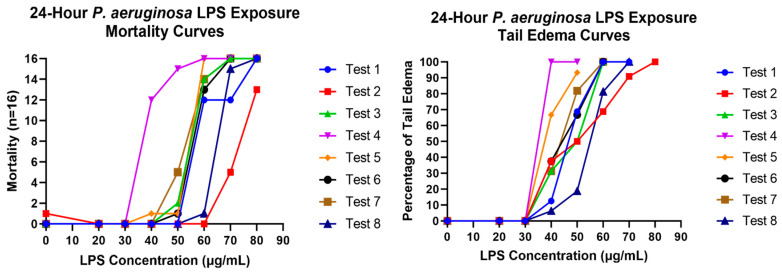
LPS concentration response curves for replicate tests during the optimization phase of assay development. The left graph depicts the percentage of fish with tail edema in response to increasing *P. aeruginosa* LPS concentrations. The right graph depicts the number of dead fish in response to increasing *P. aeruginosa* LPS concentrations. The goal was to determine a concentration that yielded close to 50% mortality while eliciting 100% combined tail edema and mortality in all fish. A concentration of 60 µg/mL of *P. aeruginosa* LPS was chosen for use in follow-on chemical screening.

*P. aeruginosa* LPS endpoint screening optimization was conducted to select a single LPS concentration that would produce a consistent endotoxic effect to facilitate reliable and repeatable drug compound screening studies. The goal was to obtain near 50% mortality with 24-h LPS exposed fish while generating a 100% combined response of tail edema and mortality. Figure 1 shows an example of a mild tail edema indictive of LPS endotoxicity in larval zebrafish. Figure 3 shows LPS concentration response curves for tail edema and mortality during the protocol optimization and reference compound studies. Based on these response data, a concentration of 60 µg/mL *P. aeruginosa* LPS was selected to elicit mortality and tail edema responses suitable for demonstrating the rescue potential of candidate drugs. While the ROS endpoint did show a quantifiable and statistically significant concentration response to increasing concentrations of LPS (see Supplemental Data), it was not included as a drug screening endpoint. The additional time required for ROS measurements could not be justified given that mortality and tail edema together provided reliable and reproducible data sufficient for targeting efficacious drugs to prevent or treat sepsis.

### 3.2. Model Compound Testing

Four model compounds (fasudil, hydrocortisone, dexamethasone, and fisetin) identified from prior published zebrafish LPS studies were evaluated. There was little change in LPS LC50 values with and without fisetin. LC50 values increased to 78.2 (95% confidence limits 72.6–84.3 µg/mL) from 73.6 µg/mL (95% confidence limits 68.7–78.8 µg/mL) with and without fisetin (400 µM) respectively and to 61.8 (95% confidence limits 57.4–66.5 µg/mL) from 60.5 µg/mL (95% confidence limits 56.3–65.0 µg/mL) with and without fasudil (10 µM). Neither compound showed complete rescue in any of the exposed fish at the 60 µg/mL LPS challenge concentration, although fasudil did show a statistically significant (*p* = 0.0233) reduction in mortality at 24 h post exposure. Prior work with fisetin by Molagoda et al. (2021) [31] and other work by Hsu et al. (2018) [30] used LPS microinjection methods and mortality rates in LPS positive controls for those studies were 30% (after 36 h) and 5% (after 24 h) respectively. The LPS challenge used in this study led to >60% mortality in LPS positive controls, so direct comparisons may not be appropriate given the differing exposure route and increased endotoxic challenge. Likewise, for fasudil, our selection of LPS (*P. aeruginosa* rather than *E. coli*) differed from prior work and may have been responsible for some of the differences in efficacy when compared to our study. For dexamethasone, the preliminary 6-h post LPS-exposure reading demonstrated a statistically significant (*p* = 0.0091) reduction in tail edema and mortality compared to the LPS control at 60 µg/mL. However, at 24-h post LPS-exposure, there was greater mortality with dexamethasone exposure, and there was no longer statistically significant rescue from tail edema or mortality. The LPS 24-h LC50 values were 48.2 µg/mL (95% confidence limits 45.75–50.68 µg/mL) with dexamethasone (100 µM) and 54.5 µg/mL (95% confidence limits 52.21–56.96 µg/mL) without dexamethasone. Hydrocortisone showed a similar response, with marginal mortality rescue noted at 6 h post LPS exposure but with increased mortality at 24 h post LPS exposure (see Supplemental Data). No statistically significant difference was observed for LPS-exposed embryos treated with hydrocortisone.

### 3.3. Initial Drug Screening

**Figure 4:**
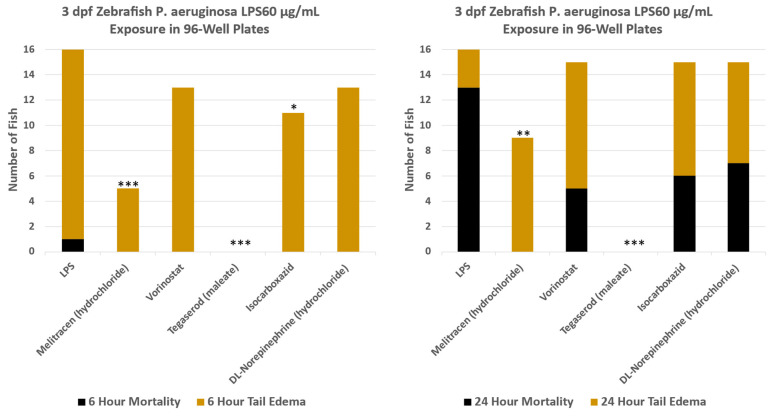
Example of the rescue results from the initial drug screening from a single 96-well plate. Tegaserod showed complete rescue from LPS-induced tail and edema and mortality (n = 16 for each test condition). * = *p* ≤ 0.05, ** = *p* ≤ 0.01, *** = *p* ≤ 0.001.

Screening of the drug library of 644 compounds yielded 80 compounds that had a statistically significant reduction (*p* = 0.05) in tail edema and mortality. To further down select top rescue drugs, a threshold of >60% rescue of tail edema and mortality was chosen. A total of 29 compounds met this threshold. Statistical significance ranged from *p* = 0.0021 to <0.0001 for these drugs. Four of the 29 compounds provided 100% rescue of tail edema and mortality: Ketanserin, Tegaserod, Brexpiprazole, and Dapoxetine. Figure 4 shows the response of Tegaserod along with other drugs from the initial screening library that were tested on the same 96 well plate. For this test plate, Tegaserod was the only drug that showed complete protection from LPS-induced tail edema and mortality (*p*-value < 0.0001). A summary of the full 644 compound screen efficacy results is provided in Supplemental Data.

Of the 29 compounds that exceeded 60% rescue, 19 came from the coagulation/anti-coagulation pathway library, eight came from the mitochondria-targeted pathway library, and two each were from the cytokine targeted pathways and the highly selective targeted pathways. Two of the 29 top compounds were classified in more than one library. Sunitinib was in the mitochondria targeted library but also in the cytokine targeted library. Iproniazid was in the mitochondria target library as well as the highly selective library. Hit rates within a library were highest for the coagulation/anti-coagulation and the mitochondria-targeted pathway libraries (Table 1).

### 3.4. Top Rescue Drug Confirmatory Testing and Down Selection

Confirmatory testing of the top 29 compounds showing >60% rescue in the initial drug screening effort yielded 16 compounds that demonstrated >80% rescue using 5 dpf larvae, with statistical significance ranging from *p* = 0.0038 to <0.0001. Five of the 16 compounds showed 100% rescue of tail edema and mortality at 5 dpf (Ketanserin tartrate, Tegaserod, Brexpiprazole, Sunitinib and Paliperidone palmitate). (See Supplemental Data for a full summary).

**Figure 5:**
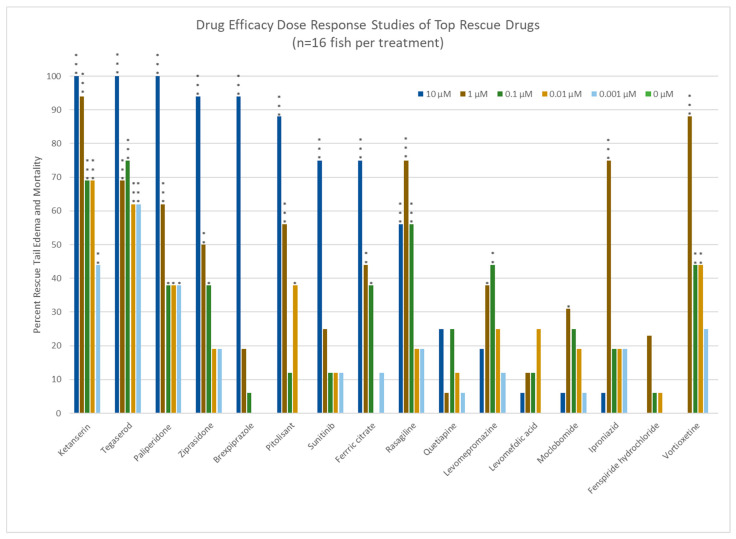
Drug efficacy concentration range response curves for the top 16 rescue drugs. * = *p* ≤ 0.05, ** = *p* ≤ 0.01, and *** = *p* ≤ 0.001. n = 16 for each test condition.

Full drug efficacy concentration-response curves for the top 16 compounds are shown in Figure 5. Ketanserin and Tegaserod showed >60% rescue at drug concentrations as low as 0.01 µM, providing the widest therapeutic window when compared to other top rescue compounds, none of which had similar rescue below 1 µM.

Further evaluation of the top 16 rescue compounds used a 24-h drug pre-treatment prior to 24-h LPS exposure. Because of the increased LPS exposure in the pre-treatment dosing studies, there was increased LPS toxicity, leading to 100% mortality in the LPS positive controls. Drug pre-treatment generally did not improve rescue for the top drugs, with none showing statistically significant improvement with drug pre-treatment (Table 2). While drug pre-treatment generally did not improve rescue capacity, it also did not alter the efficacy, which provides some early evidence that prophylactic use might be possible. Light–dark neurobehavioral testing (see Supplemental Data) identified three drugs (Ketanserin, Tegaserod, and Brexpiprazole) that showed little to no behavioral effects. The only significant difference noted was with Brexpiprazole, which showed hyperactivity (*p* = 0.005) during the dark phase with LPS exposure without 24-h drug pre-treatment, but was not statistically significant when given prophylactically 24 h prior to LPS exposure. Ketanserin, Tegaserod, and Brexpiprazole were further challenged with a lethal concentration of *K. pneumoniae* LPS (45 µg/mL). Both Brexpiprazole and Tegaserod showed 100% rescue of tail edema and mortality, while Ketanserin showed 88% rescue. Thus, these drugs show strong efficacy against endotoxicity not only from *P. aeruginosa* LPS, but also from *K. pneumoniae* LPS.

## 4. Discussion

Much has been published highlighting the role of cytokines and inflammatory pathways in the initiation and progression of sepsis. Although it was anticipated that the cytokine targeted pathways would yield a high level of positive hits for candidate sepsis rescue drugs, these pathways had the lowest number of hits of the targeted pathways screened in this study. Given the lack of efficacy of known anti-inflammatory drugs evaluated, this may not be an unexpected result. Likewise, modification of multiple cytokines might be required to affect sepsis progression, or cytokine pathways may be downstream of the true molecular initiation events that manifest in sepsis, septic shock, and death. Eight of the 16 compounds demonstrating >80% rescue with 5 dpf larvae are known to target neuromodulatory pathways. Serotonin (5-HT receptors) pathway activity, in particular involving 5-HT2a, was present as a potential target in all 8 drugs, but some also had activity within adrenergic, dopaminergic, muscarinic, and histaminergic pathways. Three compounds are monoamine oxidase inhibitors, and three other top hits targeted potassium voltage-gated channels, apoptosis regulation, and tyrosine kinase inhibition. Current drug indications for the top 16 identified sepsis rescue drugs include five antipsychotics, four antidepressants, and single drugs identified for Parkinson’s disease, irritable bowel syndrome, anti-cancer/vascular endothelial and platelet growth factors, narcolepsy, respiratory disease, folate deficiency and iron supplementation/phosphate binder.

Of the top five rescue drugs (Table 3), only Ketanserin has been previously evaluated in a clinical trial as a potential sepsis therapy. In a 10-person sepsis clinical trial, Ketanserin improved microcirculation, but its vasodilatory activity may have hindered its transition to the clinic for the treatment of sepsis [42]. Ketanserin may still have utility in situations where preventive treatment can be initiated early, prior to sepsis-associated hypotension. Except for Sunitinib, all five top rescue drugs have serotonergic (5-HT) receptor activity, which may indicate a potential common pathway for sepsis treatment and/or prevention. While Tegaserod, Brexpiprazole and Ziprasidone also target 5-HT2a receptors (and others), these drugs appear to lack the hypotension liability (as observed with Ketanserin), and thus potentially offer new options for sepsis prevention and treatment.

In addition to its well-known role within the central nervous system (CNS), 5-HT is of critical importance throughout the body, with complex modulatory roles in immune function and cellular signaling [43,44,45,46]. 5-HT receptors also play a critical role in the cardiovascular system and in the regulation of endothelial barrier function and signaling; disruption of these functions has been implicated in the progression of sepsis [47,48,49]. Further, prior work using a cecal ligation and puncture sepsis mouse model demonstrated that 5-HT proficient mice had a significantly lower survival rate than 5-HT-deficient mice [44], indicating that 5-HT pathways are likely involved in sepsis mortality. The known 5-HT activity of the discovered top rescue drugs, coupled with their ability to reduce LPS-induced vascular damage and mortality in the zebrafish endotoxemia model of sepsis, may indicate an ability to mediate endothelial function, which may be critical for silencing the signaling process involved in the progression of sepsis.

Of the 644 drugs in the screening library, a total of 103 drugs have known 5-HT receptor activity. Of these, only Ketanserin and Granisetron have been previously evaluated in clinical trials for sepsis. The Granisetron trial failed to improve 28-day mortality over placebo in a 150-person sepsis patient study [45]. Granisetron is a 5HT3 receptor antagonist and differs in 5HT receptor type from the top rescue drugs discovered through the zebrafish endotoxemia sepsis model screening efforts. Granisetron showed protection against polymicrobial sepsis-induced acute lung injury in mice [50] and sepsis-induced liver damage in rats [51]. In zebrafish, Granisetron showed a significant reduction in mortality but did not rescue from vascular damage (tail edema) and thus was not selected as a leading sepsis drug candidate for further development. Recruitment for a sepsis clinical trial is currently underway for Ondansetron, another 5HT3 receptor antagonist included in the drug screening library that was not efficacious in the zebrafish endotoxicity sepsis model.

Some results from this zebrafish sepsis model drug screening effort can be compared with findings from murine clinical studies. Published mouse LPS-induced sepsis studies showed that ciprofloxacin provided significant rescue from mortality [52]. In the zebrafish endotoxemia model used here, ciprofloxacin also provided rescue from mortality, but did not elicit rescue from vascular damage. Clinical trials for sepsis using anti-inflammatory drugs to date have not been successful [53]. Similarly, corticosteroids such as dexamethasone, methylprednisolone, and hydrocortisone showed rescue early in the zebrafish sepsis model (6-h exposure time point) but failed to show any level of rescue by 24 h post exposure.

The zebrafish LPS model has limitations. One is the use of LPS rather than an active bacterial infection to initiate a response—an issue that is shared with mammalian LPS models. In his review of animal models of sepsis, Fink (2014) notes that endotoxemia activates the innate immune system with deleterious effects, so that an intervention that blunts an inflammatory response will be beneficial, but in patients with sepsis caused by an infection, the immune response induced by microbes would be different and may have a variety of positive and negative effects [54]. As noted above, zebrafish bacterial infection models are available (e.g., Nogaret et al., 2021) [23], but they would not have the high throughput potential of the zebrafish LPS model. Other zebrafish LPS model disadvantages [55] include dose control and non-specific inflammatory responses. He et al. suggested that available gene editing technology could create genetically modified zebrafish that would better mimic human sepsis responses [55].

Despite these limitations, the zebrafish LPS model provides a reproducible approach to evaluate and identify sepsis therapy candidates, with the additional benefit of a higher throughput compared to mammalian models. The high percentage of neuromodulatory drugs identified with this rapid screen was unanticipated and warrants further exploration. Neuromodulatory drugs may be viable targets for sepsis therapy. Candidates targeting 5-HT receptors have modulatory roles in the CNS but can also affect the cardiovascular system. Other identified complementary and novel targets also merit further study as sepsis therapeutics. The leading drug candidates identified in this study exceeded the therapeutic potential of commonly used compounds such as anti-inflammatory drugs. It will be exciting for future work to evaluate the effects of the leading drug candidates in live-bacteria challenge models that more broadly emulate and induce sepsis conditions, including the use of both zebrafish and mammalian models. It will also be valuable to consider extending our data to evaluation in microphysiological systems (i.e., organ-on-a-chip) using human/patient derived tissues that could serve as a valuable in vitro drug screening platform to identify host-directed sepsis countermeasures using representative human systems [56,57], with the benefit of exploring other sepsis-related biomarkers (i.e., transcriptomic, proteomic, and metabolomic profiles).

## Figures and Tables

**Table 1:** Hit rates for compound libraries tested.

Compound Library	Number of Compounds Evaluated	Number of Compounds Exceeding 60% Rescue	Hit Rate (>60% Rescue)
MedChemExpress Coagulation and Anti-Coagulation Targeted Pathways (FDA approved only)	295	19	6.4%
MedChemExpress Mitochondria-Targeted Pathways (FDA approved only)	136	8	5.8%
Selleckchem Cytokine Targeted Pathways (FDA approved only)	140	2	1.4%
Selleckchem Highly Selective Targeted Pathways (FDA approved only)	114	2	1.7%

Note: Only 644 compounds were tested (not 685) as duplicates between pathway libraries were not tested.

**Table 2:** Effects of drug pre-treatment on 24-hour tail edema and mortality following LPS exposure.

Compound Name	24-Hour Tail Edema n = 16 fish		24-Hour Mortality n = 16 fish		24-Hour Mortality and Tail Edema n = 16 fish	
	No Drug Pre-Treatment	Drug Pre-Treatment	No Drug Pre-Treatment	Drug Pre-Treatment	No Drug Pre-Treatment	Drug Pre-Treatment
Ketanserin (tartrate)	0	0	0	0	0	0
Ketanserin	0	0	0	0	0	0
Brexpiprazole	1	0	0	1	1	1
Tegaserod (maleate)	1	0	1	0	2	0
Sunitinib (Malate)	4	1	1	1	5	2
Sunitinib	4	1	3	3	7	4
Ziprasidone (hydrochloride monohydrate)	2	0	5	4	7	4
Paliperidone palmitate	3	2	6	10	9	12
Ziprasidone	2	4	8	6	10	10
Pitolisant	2	0	13	16	15	16
Ferric citrate	2	0	13	15	15	15
Fenspiride (hydrochloride)	0	0	16	16	16	16
Levomefolic acid	1	0	15	16	16	16
Levomepromazine	1	0	15	16	16	16
Rasagiline (mesylate)	0	0	16	16	16	16
Vortioxetine	0	0	16	16	16	16
Quetiapine (hemifumarate)	1	1	15	15	16	16
Moclobemide	0	0	16	16	16	16
Iproniazid	1	0	15	16	16	16

Heatmap of combined 24-hour mortality and tail edema represents relative rescue of top drug candidates with green indicating full rescue and red indicating no rescue from tail edema and mortality.

**Table 3:** Summary results, receptor targets, and drug owners for top five rescue drugs.

	No Pre-Treatment Rescue (n = 16)	Pre-Treatment Rescue (n = 16)	Post Test Behavioral Assay	Known Receptor Targets	Current Drug Use Owners (Brand Name)
Ketanserin	100%	100%	Normal	5-HT2a (inverse agonist)	Jansen (Sufrexal)
Tegaserod	88%	100%	Normal	5-HT4 (partial agonist) 5-HT2a,b and c (antagonist)	Alfasigma (Zelnorm™)
Brexpiprazole	94%	94%	Normal	5-HT1a (partial agonist) 5-HT2a (antagonist) Noradenergic α1b (antagonist) Noradenergic α2c (antagonist) Dopamine D2 (partial agonist)	Otsuka America (Rexulti^®^)
Sunitinib	69%	88%	Impaired neurobehavior from incomplete rescue of endotoxicity	Inhibits multiple receptor tyrosine kinases (RTKs) Platelet-derived growth factor receptor alpha and beta, Vascular endothelial growth factor receptor 1, 2 and 3, Mast/stem cell growth factor Kit, RTKe FLT3, Macrophage colony stimulating factor 1, Hepatocyte growth factor receptor	Pfizer (Sutent)
Ziprasidone	56%	75%	Impaired neurobehavior from incomplete rescue of endotoxicity	Dopamine D2 (antagonist) 5-HT2a, c and d (antagonist) 5-HT1a (agonist)	Pfizer (Zeldox^®^)

## Data Availability

The original contributions presented in this study are included in the article/Supplementary Material. Further inquiries can be directed to the corresponding author.

## Supplementary Materials

The following supporting information can be downloaded at: https://www.mdpi.com/article/10.3390/life14121689/s1.

## References

[1] Weintrob A.C., Murray C.K., Xu J., Krauss M., Bradley W., Warkentien T.E., Lloyd B.A., Tribble D.R., Infectious Disease Clinical Research Program Trauma Infectious Disease Outcomes Study Group (2018). Early infections complicating the care of combat casualties from Iraq and Afghanistan. Surg. Infect..

[2] Snitchler C.L., Patel D.M., Stahlman S.L., Chauhan A.V., Wells N.Y., Mcquistan A.A. (2021). Sepsis hospitalizations among active component service members, US Armed Forces, 2011–2020. MSMR.

[3] Carius B.M., Bebarta G.E., April M.D., Fisher A.D., Rizzo J., Ketter P., Wenke J.C., Bebarta V.S., Schauer S.G. (2021). A retrospective analysis of combat injury patterns and prehospital interventions associated with the development of sepsis. Prehosp. Emerg. Care.

[4] Singer M., Deutschman C.S., Seymour C.W., Shankar-Hari M., Annane D., Bauer M., Bellomo R., Bernard G.R., Chiche J.-D., Coopersmith C.M. (2016). The third international consensus definitions for sepsis and septic shock (Sepsis-3). J. Am. Med. Assoc..

[5] Philip A.M., Wang Y., Mauro A., El-Rass S., Marshall J.C., Lee W.L., Slutsky A.S., dos Santos C.C., Wen X.-Y. (2017). Development of a zebrafish sepsis model for high-throughput drug discovery. Mol. Med..

[6] Bowman T.V., Zon L.I. (2010). Swimming into the future of drug discovery: In vivo chemical screens in zebrafish. ACS Chem. Biol..

[7] Barber A.E., Fleming B.A., Mulvey M.A. (2016). Similarly lethal strains of extraintestinal pathogenic *Escherichia coli* trigger markedly diverse host responses in a zebrafish model of sepsis. MSphere.

[8] Howe K., Clark M.D., Torroja C.F., Torrance J., Berthelot C., Muffato M., Collins J.E., Humphray S., McLaren K., Matthews L. (2013). The zebrafish reference genome sequence and its relationship to the human genome. Nature.

[9] Lieschke G.J., Currie P.D. (2007). Animal models of human disease: Zebrafish swim into view. Nat. Rev. Genet..

[10] Fleming A., Alderton W. (2013). Zebrafish in pharmaceutical industry research: Finding the best fit. Drug Discov. Today Dis. Models.

[11] Patton E.E., Zon L.I., Langenau D.M. (2021). Zebrafish disease models in drug discovery: From preclinical modelling to clinical trials. Nat. Rev. Drug Discov..

[12] van der Sar A.M., Appelmelk B.J., Vandenbroucke-Grauls C.M., Bitter W. (2004). A star with stripes: Zebrafish as an infection model. Trends Microbiol..

[13] Sullivan C., Kim C.H. (2008). Zebrafish as a model for infectious disease and immune function. Fish Shellfish Immunol..

[14] Allen J.P., Neely M.N. (2010). Trolling for the ideal model host: Zebrafish take the bait. Future Microbiol..

[15] Masud S., Torraca V., Meijer A.H. (2017). Modeling infectious diseases in the context of a developing immune system. Curr. Top. Dev. Biol..

[16] Torraca V., Mostowy S. (2018). Zebrafish infection: From pathogenesis to cell biology. Trends Cell Biol..

[17] Sisodia B.S., Kumar V., Singh S., Singh S., Datta S., Singh J., Siddhardha B., Dyavaiah M., Syed A. (2020). Zebra Fish Infection Model: From Pathogenesis to Therapeutics. Model Organisms for Microbial Pathogenesis, Biofilm Formation and Antimicrobial Drug Discovery.

[18] Gomes M.C., Mostowy S. (2020). The case for modeling human infection in zebrafish. Trends Microbiol..

[19] Marcoleta A.E., Varas M.A., Ortiz-Severín J., Vásquez L., Berríos-Pastén C., Sabag A.V., Chávez F.P., Allende M.L., Santiviago C.A., Monasterio O. (2018). Evaluating different virulence traits of Klebsiella pneumoniae using Dictyostelium discoideum and zebrafish larvae as host models. Front. Cell. Infect. Microbiol..

[20] Leber A.T. (2019). Development of a Bacterial Infection Model in Zebrafish Embryos with Special Focus on Colistin-Resistant Klebsiella Pneumoniae. Master’s Thesis.

[21] Clatworthy A.E., Lee J.S.-W., Leibman M., Kostun Z., Davidson A.J., Hung D.T. (2009). Pseudomonas aeruginosa infection of zebrafish involves both host and pathogen determinants. Infect. Immun..

[22] Sullivan C., Matty M., Jurczyszak D., Gabor K., Millard P., Tobin D., Kim C. (2017). Infectious disease models in zebrafish. Methods Cell Biol..

[23] Nogaret P., El Garah F., Blanc-Potard A.-B. (2021). A Novel Infection Protocol in Zebrafish Embryo to Assess Pseudomonas aeruginosa Virulence and Validate Efficacy of a Quorum Sensing Inhibitor In Vivo. Pathogens.

[24] Forn-Cuní G., Varela M., Pereiro P., Novoa B., Figueras A. (2017). Conserved gene regulation during acute inflammation between zebrafish and mammals. Sci. Rep..

[25] Underwood W., Anthony R. (2020). AVMA Guidelines for Euthanasia of Animals.

[26] CDC (2019). Antibiotic Resistance Threats in the United States, 2019. Atlanta, GA: U.S. Department of Health and Human Services, CDC. https://www.cdc.gov/antimicrobial-resistance/data-research/threats/index.html.

[27] Kiley J.L., Mende K., Beckius M.L., Kaiser S.J., Carson M.L., Lu D., Whitman T.J., Petfield J.L., Tribble D.R., Blyth D.M. (2021). Resistance patterns and clinical outcomes of *Klebsiella pneumoniae* and invasive *Klebsiella variicola* in trauma patients. PLoS ONE.

[28] Li Y., Wu Y., Wang Z., Zhang X.-H., Wu W.-K. (2010). Fasudil attenuates lipopolysaccharide-induced acute lung injury in mice through the Rho/Rho kinase pathway. Med. Sci. Monit..

[29] Wang Y., Wang X., Liu W., Zhang L. (2018). Role of the Rho/ROCK signaling pathway in the protective effects of fasudil against acute lung injury in septic rats. Mol. Med. Rep..

[30] Hsu A.Y., Gurol T., Sobreira T.J., Zhang S., Moore N., Cai C., Zhang Z.-Y., Deng Q. (2018). Development and characterization of an endotoxemia model in zebra fish. Front. Immunol..

[31] Molagoda I.M.N., Jayasingha J.A.C.C., Choi Y.H., Jayasooriya R.G.P.T., Kang C.-H., Kim G.-Y. (2021). Fisetin inhibits lipopolysaccharide-induced inflammatory response by activating β-catenin, leading to a decrease in endotoxic shock. Sci. Rep..

[32] Adadey S.M., Yakass M.B., Agyemang S., Duodu S. (2019). The Modulatory Effect of Lead Drug Candidates on Inflammatory Gene Expression in Sepsis: A Mini-Review. Curr. Drug Discov. Technol..

[33] Bergmann C.B., Beckmann N., Salyer C.E., Hanschen M., Crisologo P.A., Caldwell C.C. (2021). Potential targets to mitigate trauma-or sepsis-induced immune suppression. Front. Immunol..

[34] Dare A.J., Phillips A.R., Hickey A.J., Mittal A., Loveday B., Thompson N., Windsor J.A. (2009). A systematic review of experimental treatments for mitochondrial dysfunction in sepsis and multiple organ dysfunction syndrome. Free Radic. Biol. Med..

[35] Huerta L.E., Rice T.W. (2019). Pathologic difference between sepsis and bloodstream infections. J. Appl. Lab. Med..

[36] Karlsson J., von Hofsten J., Olsson P.E. (2001). Generating Transparent Zebrafish: A Refined Method to Improve Detection of Gene Expression During Embryonic Development. Mar. Biotechnol..

[37] Ducharme N.A., Reif D.M., Gustafsson J.-A., Bondesson M. (2015). Comparison of toxicity values across zebrafish early life stages and mammalian studies: Implications for chemical testing. Reprod. Toxicol..

[38] Basnet R.M., Zizioli D., Taweedet S., Finazzi D., Memo M. (2019). Zebrafish Larvae as a Behavioral Model in Neuropharmacology. Biomedicines.

[39] Kokel D., Dunn T.W., Ahrens M.B., Alshut R., Cheung C.Y.J., Saint-Amant L., Bruni G., Mateus R., van Ham T.J., Shiraki T. (2013). Identification of nonvisual photomotor response cells in the vertebrate hindbrain. J. Neurosci..

[40] Peng X., Lin J., Zhu Y., Liu X., Zhang Y., Ji Y., Yang X., Zhang Y., Guo N., Li Q. (2016). Anxiety-related behavioral responses of pentylenetetrazole-treated zebrafish larvae to light-dark transitions. Pharmacol. Biochem. Behav..

[41] Hamilton M.A., Russo R.C., Thurston R.V. (1977). Trimmed Spearman-Karber Method for Estimating Median Lethal Concentrations in Toxicity Bioassays. Environ. Sci. Technol..

[42] Vellinga N.A., Veenstra G., Scorcella C., Koopmans M., van Roon E.N., Ince C., Boerma E.C. (2015). Effects of ketanserin on microcirculatory alterations in septic shock: An open-label pilot study. J. Crit. Care.

[43] Ahern G.P. (2011). 5-HT and the immune system. Curr. Opin. Pharmacol..

[44] Zhang J., Bi J., Liu S., Pang Q., Zhang R., Wang S., Liu C. (2017). 5-HT Drives Mortality in Sepsis Induced by Cecal Ligation and Puncture in Mice. Mediat. Inflamm..

[45] Guan J., Liao Y., Guo Y., Yu S., Wei R., Niu M., Gan J., Zhang L., Li T., Lv J. (2022). Adjunctive granisetron therapy in patients with sepsis or septic shock (GRANTISS): A single-center, single-blinded, randomized, controlled clinical trial. Front. Pharmacol..

[46] Huang Y., Ji Q., Zhu Y., Fu S., Chen S., Chu L., Ren Y., Wang Y., Lei X., Gu J. (2022). Activated platelets autocrine 5-hydroxytryptophan aggravates sepsis-induced acute lung injury by promoting neutrophils extracellular traps formation. Front. Cell Dev. Biol..

[47] Ramage A.G., Villalón C.M. (2008). 5-hydroxytryptamine and cardiovascular regulation. Trends Pharmacol. Sci..

[48] Tanaka T., Mori M., Sekino M., Higashijima U., Takaki M., Yamashita Y., Kakiuchi S., Tashiro M., Morimoto K., Tasaki O. (2021). Impact of plasma 5-hydroxyindoleacetic acid, a serotonin metabolite, on clinical outcome in septic shock, and its effect on vascular permeability. Sci. Rep..

[49] Neumann J., Hofmann B., Dhein S., Gergs U. (2023). Cardiac roles of serotonin (5-HT) and 5-HT-receptors in health and disease. Int. J. Mol. Sci..

[50] Wang J., Gong S., Wang F., Niu M., Wei G., He Z., Gu T., Jiang Y., Liu A., Chen P. (2019). Granisetron protects polymicrobial sepsis-induced acute lung injury in mice. Biochem. Biophys. Res. Commun..

[51] Aboyoussef A.M., Mohammad M.K., Abo-Saif A.A., Messiha B.A. (2021). Granisetron attenuates liver injury and inflammation in a rat model of cecal ligation and puncture-induced sepsis. J. Pharmacol. Sci..

[52] Ogino H., Fujii M., Ono M., Maezawa K., Kizu J., Hori S. (2009). In vivo and in vitro effects of fluoroquinolones on lipopolysaccharide-induced pro-inflammatory cytokine production. J. Infect. Chemother..

[53] Ulloa L., Brunner M., Ramos L., Deitch E.A. (2009). Scientific and clinical challenges in sepsis. Curr. Pharm. Des..

[54] Fink M.P. (2014). Animal models of sepsis. Virulence.

[55] He J., Xu P., Chen R., Chen M., Wang B., Xie Y., Yang Q., Sun D., Ji M. (2024). Exploiting the zebrafish model for sepsis research: Insights into pathophysiology and therapeutic potentials. Drug Des. Dev. Ther..

[56] Liang N.W., Wilson C., Davis B., Wolf I., Qyli T., Moy J., Beebe D.J., Schnapp L.M., Kerr S.C., Faust H.E. (2024). Modeling lung endothelial dysfunction in sepsis-associated ARDS using a microphysiological system. Physiol. Rep..

[57] Liu D., Langston J.C., Prabhakarpandian B., Kiani M.F., Kilpatrick L.E. (2024). The critical role of neutrophil-endothelial cell interactions in sepsis: New synergistic approaches employing organ-on-chip, omics, immune cell phenotyping and in silico modeling to identify new therapeutics. Front. Cell. Infect. Microbiol..

